# Multiple Treatment Cycles of Neural Stem Cell Delivered Oncolytic Adenovirus for the Treatment of Glioblastoma

**DOI:** 10.3390/cancers13246320

**Published:** 2021-12-16

**Authors:** Jennifer Batalla-Covello, Hoi Wa Ngai, Linda Flores, Marisa McDonald, Caitlyn Hyde, Joanna Gonzaga, Mohamed Hammad, Margarita Gutova, Jana Portnow, Tim Synold, David T. Curiel, Maciej S. Lesniak, Karen S. Aboody, Rachael Mooney

**Affiliations:** 1 Department of Developmental and Stem Cell Biology, City of Hope, Duarte, CA 91010, USA; jcovello@coh.org (J.B.-C.); gngai@coh.org (H.W.N.); lflores@coh.org (L.F.); mmcdonald@coh.org (M.M.); chyde@coh.org (C.H.); jgonzaga@coh.org (J.G.); mhammad@coh.org (M.H.); mgutova@coh.org (M.G.); 2 Department of Medical Oncology, City of Hope, Duarte, CA 91010, USA; jportnow@coh.org; 3 Department of Cancer Biology, City of Hope, Duarte, CA 91010, USA; tsynold@coh.org; 4 Department of Radiation Oncology, Washington University School of Medicine, St. Louis, MO 63110, USA; dcuriel@wustl.edu; 5 Department of Neurological Surgery, Northwestern University, Chicago, IL 60611, USA; Maciej.lesniak@northwestern.edu

**Keywords:** glioma, oncolytic virus, immunotherapy, stem cell carrier, neural stem cell, viral delivery

## Abstract

**Simple Summary:**

The human body’s ten trillion cells are constantly assailed with environmental insults and genetic susceptibilities that can initiate tumor formation. Yet, most people live cancer-free for decades. This bewildering feat is due, in part, to the remarkable ability of our immune system to recognize and eliminate tumor cells. Unfortunately, 12,000 Americans/year are diagnosed with a rare, aggressive, and fatal tumor that escapes immune recognition: glioblastoma. Here, we continue efforts to develop a treatment capable of stimulating immune recognition of glioblastoma. The treatment is based on an oncolytic virus that causes tumor-selective infections. Neural stem cells are used to enhance viral distribution throughout the tumor. This study selects a dosing strategy to enable more comprehensive viral inoculation of the tumor than was possible in our previous clinical trial. This research demonstrates to the broader community that multiple-cycle oncolytic virotherapy may be therapeutically beneficial despite an anti-viral response after the first administration.

**Abstract:**

Tumor tropic neural stem cells (NSCs) can improve the anti-tumor efficacy of oncovirotherapy agents by protecting them from rapid clearance by the immune system and delivering them to multiple distant tumor sites. We recently completed a first-in-human trial assessing the safety of a single intracerebral dose of NSC-delivered CRAd-Survivin-pk7 (NSC.CRAd-S-pk7) combined with radiation and chemotherapy in newly diagnosed high-grade glioma patients. The maximum feasible dose was determined to be 150 million NSC.CRAd-Sp-k7 (1.875 × 10^11^ viral particles). Higher doses were not assessed due to volume limitations for intracerebral administration and the inability to further concentrate the study agent. It is possible that therapeutic efficacy could be maximized by administering even higher doses. Here, we report IND-enabling studies in which an improvement in treatment efficacy is achieved in immunocompetent mice by administering multiple treatment cycles intracerebrally. The results imply that pre-existing immunity does not preclude therapeutic benefits attainable by administering multiple rounds of an oncolytic adenovirus directly into the brain.

## 1. Introduction

Oncolytic virotherapy is a promising approach for treating drug- or radiation-refractory brain cancer. Oncolytic viruses (OV) kill tumor cells directly via oncolysis; and also indirectly, by stimulating anti-tumor immune responses. Among oncolytic viral species used in clinical trials, adenoviruses possess several advantageous properties, including relatively simple genetic modification, inherent immunogenicity, and high viral titer production [1]. To date, OVs from nine different families, including both DNA and RNA viruses, have been successfully transitioned from preclinical studies into 31 early-phase clinical trials in patients with brain tumors. Although a robust survival benefit remains to be shown in larger, randomized phase II/III trials, seven phase I/II trials have demonstrated remarkable tumor regressions in isolated patients. One reason that positive responses were not observed in the majority of OV-treated patients may be that optimal colonization of the tumor by the virus was limited by immune inactivation of the virus. Additionally, there was likely poor viral access to scattered infiltrative GBM cells that were separated from main tumor mass by normal tissue [2,3,4,5].

Neural stem cells (NSCs) have the intrinsic capability to migrate to invasive primary and secondary brain tumor sites in various preclinical models [6], whether delivered intracranially in the opposite hemisphere, the lateral ventricle, or intravenously [7]. NSCs can be used to deliver anti-cancer agents, including oncolytic adenoviruses, specifically to brain tumors [8]. Their use results in several advantages, including (1) protection of the virus from immune inactivation on route to tumor sites; (2) improved tumor penetration and distribution; and (3) an ability to carry virus across normal tissue to seed distant invasive tumor foci [9]. 

Our group uses NSCs as a delivery vehicle to improve oncolytic viral delivery, with a strong focus on a particular virus with potent anti-GBM activity (CRAd-S-pk7) [9]. We have previously demonstrated that NSC-mediated CRAd-S-pk7 delivery provides therapeutic added value when treating human glioma xenografts in immunodeficient mice [10,11]. Most recently, the clinical safety of CRAd-S-pk7-transduced NSCs (NSC.CRAd-S-pk7) was demonstrated in a first-in-human study in newly diagnosed high-grade glioma patients. The highest administered dose was a single, intracavitary injection of 150 million NSC.CRAd-S-pk7s (1.875 × 10^11^ Viral Particles) [12,13]—the maximum dose deliverable within a single infusion due to volume limitations for intracerebral administration [13]. Further dose escalation may be safe and provide additional efficacy benefits [14].

Here, we report IND-enabling data supporting the use of multiple administrations. A concern with repeated OV dosing is the inactivation of the virus due to the emergence of neutralizing antibodies [15]. Indeed, in the first-in-human study, anti-Ad5 neutralizing antibodies were detected in participants’ blood within a week after administration of the single intracerebral dose of NSC-CRAd-S-pk7 [13]. However, it is unclear if these neutralizing antibodies were able to eliminate the OV. We hypothesize that, by being packaged within NSCs, CRAd-S-pk7 will be protected from destruction by neutralizing antibodies and complement while being transported to tumor foci by the NSCs. Here, we test this hypothesis and investigate the therapeutic advantage of multiple administrations within both immunodeficient and immunocompetent mouse models.

## 2. Materials and Methods

Tumor cell culture. All cell lines were cultured in Dulbecco’s Modified Eagle’s Medium (DMEM) (Invitrogen, Waltham, MA, USA) supplemented with 10% fetal bovine serum (Gemini Bio, West Sacramento, CA, USA), 1% l-glutamine (Invitrogen), and 1% penicillin/streptomycin (Invitrogen), and maintained at 37 °C in a humidified incubator (Thermo Electron Corporation, Waltham, MA, USA) containing 6% CO_2_. Cells were passaged when they reached 80% confluency using a 0.25% trypsin/EDTA solution (Invitrogen); media wase changed every 2–3 days. U251.eGFP was provided by Christine Brown. The GL261.ffluc line used for this study was a murine glioma cell line of C57BL/6J origin further modified in Benham Badie’s laboratory (City of Hope, Duarte, CA, USA) to stably express firefly luciferase. He generously provided some frozen vials to Aboody’s laboratory (City of Hope, Duarte, CA, USA). Wild-type GL261 cells express low levels of MHC Class 1, but not class II molecules, and express some costimulatory molecules, resulting in a classification as moderately immunogenic.

In vitro verification of CRAd-S-pk7 tumor lysis. Tumor cells were plated at 5 × 10^5^ cells per well in 6-well plates 24 h prior to exposure to CRAd-S-pk7 (MOI = 10). Incucyte software was utilized to capture time-elapsed photos based on the phase contrast channel over a period of 3 days.

Neural stem cells. The v-myc-immortalized human HB1.F3.CD21 NSC line (approved by the Food and Drug Administration for human clinical trials via local injection, Identifier: NCT01172964) was obtained from Seung Kim (University of British Columbia, Vancouver, BC, Canada). Permission to use fetal tissue was granted to S. U. Kim (University of British Columbia, Vancouver, BC, Canada) by the University of British Columbia Clinical Research Screening Committee for Studies Involving Human Subjects. Tissue was obtained from the Anatomical Pathology Department of Vancouver General Hospital. The HB1.F3 immortalized human NSC line was derived from primary cultures of fetal telencephalon (15 weeks gestation) by immortalization with an amphotropic, replication-incompetent retrovirus with the v-myc gene [16,17,18]. Clones were isolated, expanded, and designated as HB1 NSC lines [17,19]. One of these clones, HB1.F3, was transduced with the retroviral vector pMSCV-puro/CD, and clones were then isolated and expanded. HB1.F3.CD clone 21 was given to the City of Hope under a Material Transfer Agreement.

Production of HB1.F3.CD21.CRAd-S-pk7 cell banks. The research-grade clinical equivalent NSC-CRAd-S-pk7 cell banks (Banks 2 and QB53) were manufactured and release-tested in the Aboody Lab (City of Hope, Duarte, CA, USA). For bank 2, one vial of HB1.F3.CD21 (NSC) passage 26 from Quantum Bank 30 was thawed at 37 °C and plated in T-182 flasks (Genessee Scientific, San Diego, CA, USA) at 2 × 10^4^/cm^2^. Cells were cultured in complete growth media (DMEM supplemented with 10% Fetal Bovine Serum and 1% GlutaMAX™) and were incubated at 37 °C and 6% CO_2_. NSCs were passaged twice post-thaw by washing with 1 × DPBS (without calcium and magnesium) and detaching with 0.25% Trypsin/EDTA (Gemini). A representative T-182 flask was harvested for a cell count of NSCs per flask. NSCs were transduced with Master Viral Seed Stock CRAd-S-pk7 (Batch #0806-349-0001-1) at a multiplicity of infection (MOI) of 50 with a titer of 6.7 × 10^10^ IFU/mL. After a 2-h incubation, a total yield of 2.6 × 10^8^ viable cells was harvested with 97% viability. Cells were vialed at 8.5 × 10^6^ cells/mL in cryopreservation medium (Cryostor 10), frozen in a Cryo1 °C Freezing Container (Nalgene) with a cooling rate of −1 °C/min in a −80 °C freezer, and then transferred to vapor phase of liquid nitrogen for long-term storage.

For bank 53, three vials of HB1.F3.CD21 (NSC) passage 25 from Quantum Bank 50 were thawed at 37 °C and plated into a fibronectin-coated fiber bioreactor, as previously described [20]. Cells were expanded for seven days, then transduced with CRAd-S-pk7 (MOI = 27). One hour after adding the virus, media were exchanged, and cells were harvested for freezing. A separate bank (QB51, MOI = 14.6, 1 h freeze down) was made using a CRAd-S-pk7 modified to express firefly luciferase (Vector Biolabs, Malvern, PA, USA).

Characterization of HB1.F3.CD21.CRAd-S-pk7 cell banks. All NSC.CRAd-S-pk7 cells were release-tested and characterized as >90% viable, >80% recovered post-thaw, >95% nestin +, and free of mycoplasma. Infectious viral load per NSC was determined using a commercially available titer kit per manufacturer’s instructions (Adeno-X from Takara). Viability was determined using a fluorescent permeability dye (Viacount, Luminex, Austin, TX, USA), and lysis time was monitored via time-lapse photography (Incucyte, Essenbioscience, Newark, UK). Surface Hexon Expression on transduced NSCs was quantified by flow cytometry using standard procedures. Briefly, the transduced NSCs were washed with PBS (with FBS and Sodium Azide). A solution containing both fixation and permeabilization reagents (Fix and Perm, Life Technologies, Carlsbad, CA, USA) was then added to treat the cells, and the cells were then re-washed. Anti-hexon (MAB 8052, Millipore-Sigma, Burlington, MA, USA) was then added and incubated for 30 min. After additional washing, an Alexa fluor-conjugated antimouse IgG secondary antibody was added (SAB4600388, Millipore-Sigma). Positive cells were then assessed using flow cytometry (Guava EasyCyte HT, Luminex).

In vivo orthotopic glioma models. Mice were maintained under specific pathogen-free conditions in the City of Hope Animal Resource Center, an AAALAC-accredited facility. All procedures were reviewed and approved by the City of Hope Animal Care Committee. For the immunocompetent models, 6–8-week-old C57BL/6J mice (weight 18–21 g) were used in this study. C57BL/6J mice are immunocompetent and can, therefore, develop effective adenoviral clearance responses. For the immunodeficient models, 6–8-week-old athymic nude (the Jackson Laboratory, Bar Harbor, ME, USA) were used because they are unable to provide mouse adaptive immune responses including: (1) CD4-dependent antibody formation; and (2) CD8-dependent killing of virus-infected or malignant cells.

Tumor implantation. On study day 0, all groups received an intraperitoneal (IP) injection of ketamine–xylazine cocktail (dose 132 mg/kg of ketamine and 8.8 mg/kg of xylazine), followed by an intracranial (IC) stereotactic injection of tumor cells (GL261.dsRed, GL261.ffluc, or U251.ffluc depending on experiment) in the right frontal lobe. Surgical coordinates were 2 mm right of bregma and 0.5 mm rostral. Tumor cells were injected at three levels (0.667 µL of tumor cells injected 2.5 mm deep, another 0.667 µL injected at 2.25 mm, and then 0.667 µL injected at 2.00 mm). The skull was sealed with bone wax and the scalp gently closed with surgical glue. Analgesia (Slow release buprenex) was administered immediately upon waking.

Final preparation of NSC.CRAd-S-pk7 study agent. On the day of treatment, frozen vials of NSC.CRAd-S-pk7 were thawed at 37 °C, and washed three times with cell wash buffer containing Perfusion Fluid CNS (PFCNS) with 2% Human Serum Albumin (HSA) by centrifugation to remove cryopreservation media. The cell pellet was re-suspended to a concentration of 5 × 10^5^ NSC.CRAd-S-pk7/4 µL (1.25 × 10^8^ cells/mL) in PFCNS/2% HSA and administered intratumorally.

Live animal imaging. To confirm this increased viral load transferred to tumor cells when NSC.CRAd-S-pk7 cells were frozen at 24 vs. 1 h, CRAd-S-pk7.ffluc signal was monitored in vivo for four days after administration via bioluminescence using the SPECTRAL Ami X imaging system. Before imaging, mice were anesthetized by isoflurane (1.5 L/oxygen, 4% isoflurane) in an induction chamber and injected IP with D-luciferin substrate suspended in PBS at 4.29 mg/mouse. Mice were maintained under anesthesia in a chamber, and 7 min after injection of luciferin, the NSCs were imaged using a charge-coupled device camera (the SPECTRAL Ami X) coupled to Ami X image acquisition and analysis software. Light emission was measured over an integration time of 300 s.

Clinical observations. For long-term survival studies, all study mice were weighed weekly and observed daily Monday through Friday for general good health, e.g., good/water intake, urine/feces production, no signs of scruffy hair coat, emaciation, or hunched posture. Any debilitating terminal criteria including misshapen skull, seizures, tremors, labored or difficult breathing, weight loss (>20% body weight), hypo- or hyperthermia, impaired ambulation, obvious illness, or inability to remain upright warranted immediate euthanasia.

Blood chemistry. In a pilot study involving immunocompetent mice (*n* = 6), terminal intracardiac blood was collected using heparinized needles and transported to the Aboody Laboratory for chemistry analysis. The blood samples were tested using the VetScan Comprehensive Diagnostic Profile, which consists of 14 analytes, to evaluate the toxicity profile of 3 rounds of NSC.CRAd-S-pk7 and 1 round of NSC.CRAd-S-pk7. See Table 1 for an explanation of each analyte.

Tumor size. In the pilot immunocompetent study (*n* = 6), two mice from each group were euthanized and their brains harvested on D28 (7 days after the last treatment). Brains were post-fixed in 10% paraformaldehyde for histopathology analysis. The tissue was sent to the City of Hope pathology core (Duarte, CA, USA), where it was processed using routine histological methods: paraffin-embedded; sectioned; mounted on slides; and stained with hematoxylin, eosin, anti-F4/80, and anti-PD1. Slides were returned to the Aboody lab and scanned via automated light microscopy (Zeiss) to visualize tumor size and immune infiltration.

NanoString analysis. Total RNA was extracted from brain quartiles containing the treated tumor (TriReagent, Sigma Aldrich, St. Louis, MO, USA), following the manufacturer’s instructions. RNA was further purified (RNeasy Mini Kit, Qiagen, Hilden, Germany), then quantified (NanoDrop-1000, Thermo Fisher, Waltham, MA, USA). Samples with RNA integrity values of >7.0 were included for gene expression analysis (NanoString nCounter, NanoString Technologies, Seattle, WA, USA). RNA (*n* = 4/gp) was analyzed using the nCounter Mouse PanCancer Immune Profiling Panel. Raw gene expression data were analyzed (nSolver v3.0.22, NanoString Technologies, Beijing, China). Pathway scores summarize data from a pathway’s genes into a single, normalized, and standardized z-scaled score. Cell profiling uses marker genes stably expressed in immune cell types to estimate relative abundance in sample groups by measuring the average log-scale expression of characteristic genes [21].

Statistical methods. Sample sizes for in vivo studies were powered based on the survival analysis observed in a pilot *n* = 6 study. A 1-sided exact log-rank test with 11 mice per group was expected give 90% power at a 0.05 significance level to detect a hazard ratio between an active group (3-rounds of NSC.CRAd-S-pk7, 1-round NSC.CRAd-S-pk7) and the control group of approximately 0.12 when the control group had a median survival time of 40 days, while the active group had a median survival time of 100 days using a 2-sided log-rank test. All other data are presented as mean ± SEM unless otherwise stated. Statistical significance (*p* < 0.05) was determined using two-tailed Student’s *t*-tests unless otherwise stated.

## 3. Results

### 3.1. Tumor Line Selection

**Figure 1:**
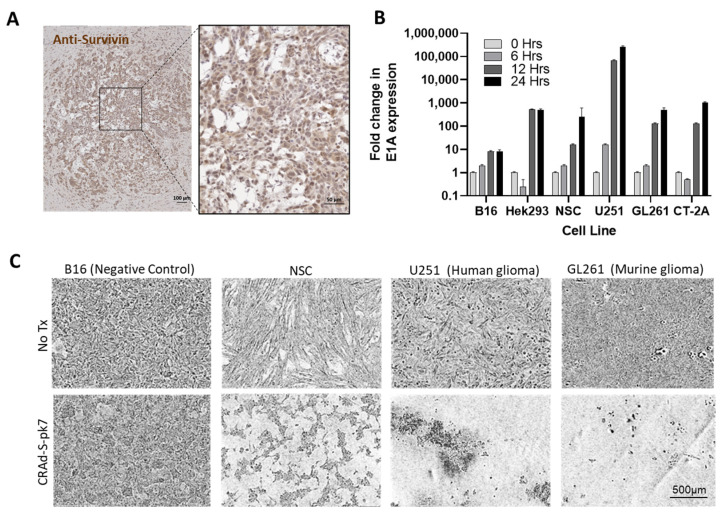
The murine GL261 cell line supports replication of the human adenovirus CRAd-S-pk7. (**A**) GL261 was engrafted in brain of C57/BL-6 immunocompetant mice. Fourteen days later, harvested brain was cryosectioned. Brightfield image is shown of brain slice stained with Anti-survivin primary, HRP-conjugated secondary, and DAB substrate. (**B**) Quantitative PCR results showing amplification of CRAd-S-pk7 specific E1A gene expression at select timepoints post-infection in both non permissive (B16); and permissive (HEK293, NSC, U251, GL261, CT-2A) cell lines. (**C**) Representative phase-contrast images acquired after three days of culture are shown. Cultures contained B16 (negative control), HB1.F3.CD21 (positive control), GL261 (murine glioma), and U251 (human glioma) cells infected with CRAd-S-pk7 at MOI 10 or no virus control. Scale bar = 500 µm and applies to all images in (**C**).

Our question requires a study within an immunocompetent syngeneic model. Adenoviruses cannot typically lyse and release new infectious particles when infecting mouse cells, including B16 cells (Figure 1) [22]. It was, thus, necessary to identify a mouse tumor line that was permissive for adenoviral replication. The murine glioma cell line, GL261, is currently accepted as the gold standard line to use when generating rodent glioma models. Here, we demonstrate that GL261 express survivin (Figure 1A) and can be infected by CRAd-S-k7 in vitro. GL261 supports CRAd-S-pk7 replication (Figure 1B), resulting in an apparent cytopathic effect (Figure 1C). While the lysis is not as extensive as that observed in human U251 glioma cells, GL261 cells can serve as a semi-permissive cell line when testing NSC.CRAd-S-pk7 efficacy in immunocompetent GL261 mouse models of glioma. We next prepared research banks of CRAd-S-pk7-transduced NSCs to be used during in vivo efficacy studies. Clinical-equivalent NSC.CRAd-S-pk7 banks were manufactured and release tested, as previously described [13].

### 3.2. Increasing NSC.CRAd-S-pk7 Dose Using Multiple Administrations

Increasing the administered volume is not a possibility, either through intratumoral injections or intraventricular, due to safety concerns [23]. We therefore considered if multiple, weekly NSC.CRAd-S-pk7 intracerebral administrations would increase therapeutic efficacy over a single dose, despite the emergence of anti-Ad5 antibodies following the first treatment. We hypothesized that NSCs could protect CRAd-S-pk7 by physically shielding the virus from serum antibodies, thus preventing initiation of the classical complement cascade that rapidly eliminates viruses. Our rationale was that even the first administration is vulnerable to complement-mediated destruction via the alternative pathway (antibody independent) [24], yet substantial therapeutic efficacy is still observed.

#### 3.2.1. NSCs Protect CRAd-S-pk7 from Serum Neutralization

**Figure 2:**
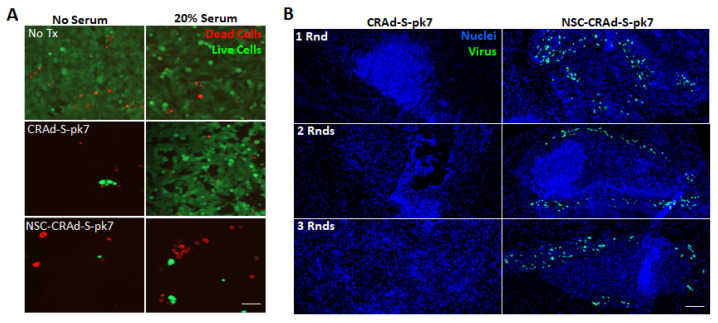
NSCs protect CRAd-S-pk7. (**A**) Representative fluorescence images of day 7 U251 brain cancer cell cultures stained with calcein-AM and ethidium bromide to visualize live (green) and dead (red) cells, respectively. Cultures were treated with either free CRAd-S-pk7 or dose-matched NSC-Crad-s-pk7 with and without the addition of 20% human serum. Scale bar *=* 50 µm and applies to all images. (**B**) Immunocompetent C57/BL-6 mice (8 weeks old females) bearing 4-day old intracranial GL261 glioma (2 × 10^3^ cells) received either 1, 2, or 3 weekly rounds of intra-tumoral CRAd-S-pk7 (2.5 × 10^7^ IU) or dose-matched NSC-CRAd-S-pk7. Brains were harvested, fixed, and cryosectioned 1 day after treatment. Brain slices were stained with anti-hexon FITC to visualize CRAd-S-pk7, and nuclei counterstained with DAPI. Scale bar *=* 100 µm and applies to all images.

The ability of complement proteins in serum to neutralize free CRAd-S-pk7 independent of pre-existing antibody presence is demonstrated both in vitro and in vivo (Figure 2). We conducted in vitro assays using cultures of U251 brain tumor cells with or without 20% human serum. Although treatment with free CRAd-S-pk7 or NSC-CRAd-S-pk7 led to tumor cell killing in the absence of human serum, only treatment with NSC-CRAd-S-pk7 led to tumor cell killing in the presence of human serum (Figure 2A), supporting our hypothesis that NSCs provide protection from complement-mediated CRAd-S-pk7 neutralization.

We also performed an in vivo experiment in immunocompetent mice in which CRAd-S-pk7 presence was visualized 1 day after administering either one, two, or three rounds of intra-tumoral treatment with either free CRAd-S-pk7 or NSC-CRAd-S-pk7. CRAd-S-pk7 transduction of GL261 glioma cells was prevented even with the first round of treatment, before the naive mouse had anti-Ad5 anti-bodies present. Furthermore, NSC-mediated CRAd-S-pk7 delivery enabled intra-tumoral CRAd-S-pk7 to be present across all three rounds of administration (Figure 2B).

#### 3.2.2. Long-Term Survival Studies

**Figure 3:**
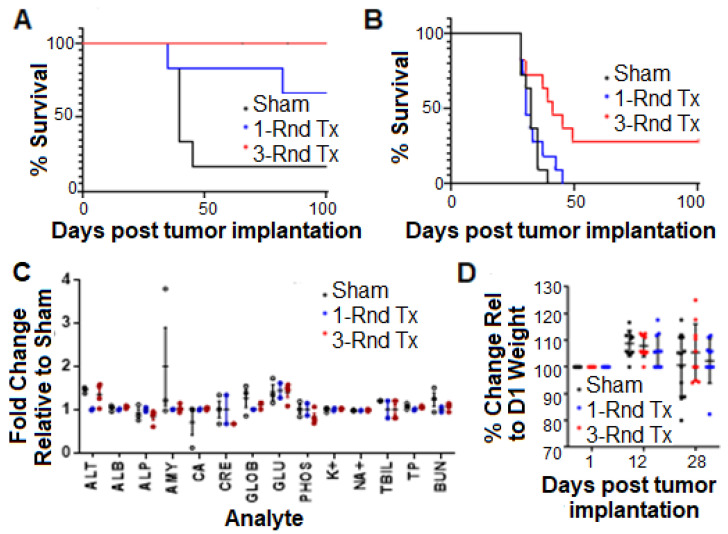
Multiple administrations of NSC-CRAd-S-pk7 prolong survival of immunocompetent mice bearing syngeneic glioma orthografts. Immunocompetent mice bearing orthotopic GL261 tumors were treated with NSC-CRAd-S-pk7 for 1 or 3 rounds. PFCNS + 2% HSA was used as a sham control. (**A**) Kaplan-Meier plot from pilot study (*n =* 5–6). (**B**) Kaplan-Meier plot from powered study (*n =* 11/group). Improved survival seen of mice treated with 3 rounds of NSC-CRAd-S-pk7 (3/11 alive at day 90) vs. 1 round (0/11 alive at day 90); *p =* 0.0067 when comparing 1 vs. 3 treatments (log-rank test). (**C**) Pilot clinical chemistry evaluation of repeated NSC.CRAd-S-pk7 administrations. On day 28, blood samples were collected from select mice in pilot study and assayed for the following parameters: albumin, alkaline phosphatase, alanine aminotransferase, aspartate aminotransferase, total bilirubin, blood urea nitrogen, calcium, phosphorus, creatine, glucose, sodium, potassium, total protein, and globulin. (**D**) Post-treatment mouse weights: each measurement is that of an individual mouse normalized to its initial weight on day 0 (W_D_/W_0_ × 100% where D is the day post-tumor injection).

Two GLP pre-clinical studies were conducted, and each was designed to compare the long-term survival of immunocompetent C57BL/6J mice inoculated orthotopically with a firefly luciferase expressing GL261 mouse glioma cell line, revealing one vs. three weekly treatments at a dose of 5 × 10^5^ NSC.CRAd-S-pk7s (Figure 3). The first was a pilot study in which mice with relatively small syngeneic orthografts (5000 GL261 cells per mouse) were randomly placed into the following treatment groups 7 days post-tumor inoculation: (1) one round of perfusion fluid central nervous system (PFCNS) + 2% human serum albumin (HSA) (control); (2) one round of NSC-CRAd-S-pk7 treatment; and (3) three weekly rounds of NSC-CRAd-S-pk7 treatment. Results showed a trend towards improved long-term survival upon administering three vs. one treatment round of NSC.CRAd-S-pk7 (Figure 3A). However, a larger study was needed to achieve statistical significance.

The pilot experiment was repeated with a study powered for statistical significance (*n =* 11/group) in a more challenging, larger tumor model (10,000 tumor cells/mouse, treatment initiated on day 12). In the powered experiment, no survival benefit was seen with a single administration of NSC-CRAd-S-pk7. The median survival was 35 days in the group receiving a single dose of NSC. CRAd-S-pk7, while the mice treated with three weekly rounds was extended to 41 days [log-rank, 3 vs. 1 Round CRAd-S-pk7 NSCs, *p =* 0.0067] (Figure 3B).

Preliminary preclinical systemic toxicity assessments (i.e., inflammation, immunodeficiency states) for multiple rounds of NSC-CRAd-S-pk7 were performed by monitoring liver and kidney enzymes using GLP standards. There was no observable difference between any of the groups (*p* > 0.05, one-way ANOVA), suggesting no obvious adverse effects of repeated NSC-CRAd-S-pk7 treatment (Figure 3C). In addition, we did not observe significant differences in body weight (Figure 3D) or in the following daily observations of mouse behavior: posture, seizures, tremors, labored breathing, and ambulation, suggesting that no gross neurological effects resulted from multiple administration of NSC-CRAd-S-pk7.

#### 3.2.3. Immunological Characterization

Interestingly, there was no survival advantage afforded by three rounds of NSC.CRAd-S-pk7 when administered to tumor-bearing nude mice, which contain innate but not adaptive immune functions (Figure S1). This suggests that repeated NSC.CRAd-S-pk7 treatment efficacy relies on the host’s adaptive immune response, not simply an increase in the administered viral load or improved viral distribution.

**Figure 4:**
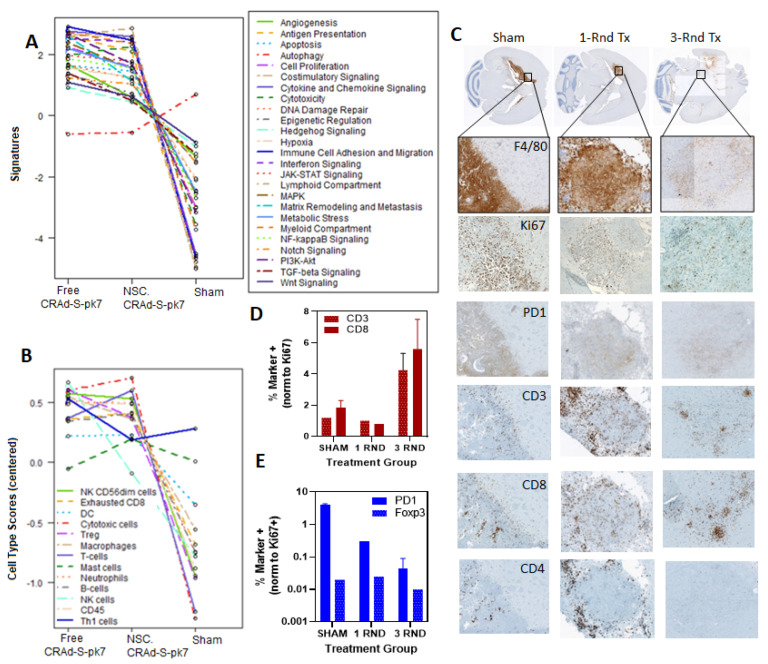
Immunological effects of NSC.CRAd-S-pk7 administration in syngeneic glioma orthograft model. (**A**,**B**) Plots depicting mean normalized expression of NanoString analysis pathway signature and cell type differentiation scores obtained using RNA isolated from C57/Bl-6 mice bearing CT-2A glioma seven days after treatment with either sham saline, CRAd-S-pk7, or dose-matched NSC-CRAd-S-pk7 (*n =* 3 per group). (**C**,**D**) Tumors were established using 5.0 × 10^3^ GL261 cells/mouse, then treated using either a Day 7 single dose or three doses of 5.0 × 10^5^ HB1.F3.CD_CRAd-S-pk7 cells on days 7, 14, and 21 (*n =* 2 per group). Twenty-eight days after the first treatment, brains were cryosectioned and processed using standard immunological techniques. Positive cell quantification was automated using ImagePro segmentation software. Results are expressed normalized with respect to Ki-67 positive cells. (**E**) Representative brain sections stained with F4/80, then 10× scanned images were tiled to showing the relative sizes of GL261 tumor in mice brains that received either sham, one round of NSC.CRAd-S-pk7, or three rounds of NSC.CRAd-S-pk7. Inset enlarged to aid visualization (10×). Sister brain sections stained with anti-Ki-67, anti-PD1, anti-CD3, anti-CD8, and anti-CD4 are shown.

The nature of the immune response that occurs after NSC-CRAd-S-pk7 treatment has not been fully established. To provide broad characterization of immune-mediated changes in the brain that occur following NSC-CRAd-S-pk7 treatment, NanoString transcriptome analysis was utilized in mice treated with a single dose of either sham saline, free CRAd-S-pk7, or NSC-CRAd-S-pk7 (Figure 4A). Brains were harvested 7 days after treatment in an effort to obtain a transcriptome snapshot when the adaptive immune response peaks (7–10 days). Substantial differences were observed in both pathway signature scores and immune infiltration scores when comparing tumors treated with either free CRAd-S-pk7 or NSC.CRAd-S-pk7 vs. Sham (Figure 4A). The only pathway signature score that remained relatively unchanged across all treatment groups was autophagy. The interpretation of how these complex changes impact overall tumor progression is still unclear, given that both suppressive and inflammatory cell types seem to be recruited. Tumor regression was not confirmed prior to harvesting brains for NanoString analysis.

To assess the spatial distribution of select T-cell subsets, two mice per group were harvested on day 28 of the pilot efficacy study, whereby immunocompetent mice were treated with a single dose either sham or NSC.CRAd-Spk7 or three weekly doses of NSC.CRAd-S-pk7. Brain slices were immunologically stained for F4/80 to find tumor progression foci and Ki-67 to identify replicating tumor cells, in addition to the following T-cell phenotypic markers (CD4+, CD3+, CD8+, Foxp3, PD1) (Figure 4).

F4/80 staining demonstrates that tumors appear noticeably smaller after one round of NSC.CRAd-S-pk7 treatment when compared to the sham control group (Figure 4E). The brains of select mice that received three treatment rounds appeared to have no tumor remaining (Figure 4E). Instead, there was a sizable tissue defect where the tumor was assumed to have engrafted, then cleared. Quantification was, therefore, performed in an adjacent tissue populated with tumor foci evident by light F4/80 staining (Figure 4C,D). These pilot data suggest that treating mice with three cycles, rather than a single cycle, may result in increased CD3 and CD8 positive tumor-localized T-cells. A decrease in PD-1 positive cells was also observed in mice treated with three cycles rather than a single cycle.

### 3.3. Discussion

Novel treatments that can improve survival for glioblastoma patients are urgently needed. NSC.CRAd-S-pk7 is a novel therapeutic product under clinical investigation, but its true therapeutic potential cannot be adequately tested until the dose-limiting volume restrictions are resolved. The results presented here motivated our decision to pursue intracerebral administration of multiple weekly doses. This approach will be clinically tested for safety in an upcoming Phase 1 trial (IND 19532).

#### 3.3.1. Multiple NSC.CRAd-S-pk7 Administration Prolongs Survival More Than a Single Dose

The most important result of these IND-enabling studies is that three weekly intracerebral rounds of a clinical-equivalent NSC.CRAd-S-pk7 dose improved the survival of brain tumor-bearing immunocompetent mice relative to a single administration. Possible mechanisms that may explain the superior efficacy of three administrations are: (1) a higher viral load more effectively competes with replicating tumor cells; (2) temporally spaced intra-tumoral injections improve NSC.CRAd-S-pk7 distribution and enable virus deposition in different and noncontiguous tumor regions [15,25,26]; and (3) anti-tumor immune responses are repeatedly stimulated by successive NSC.CRAd-S-pk7 treatments. Our observation that multiple treatment cycles afford a survival advantage that may be dependent on, rather than hindered by the presence of the adaptive immune system is slightly surprising given precedent literature describing rapid anti-viral clearance upon repeated administration of oncovirus [15]. In fact, there is no precedent for administering multiple OV doses within the GBM setting. However, pre-existing viral immunity is emerging as paradoxically beneficial in other tumor settings [27].

#### 3.3.2. Other Reports Showing Pre-Existing Viral Immunity Does Not Preclude Treatment Efficacy

Even though our result is one of only a few studies demonstrating the superiority of repeat dosing versus single-cycle OV therapy, this idea is consistent with a growing body of work, indicating that pre-existing immunity does not eliminate the therapeutic efficacy of oncolytic virotherapies, particularly with intra-tumoral administration. In fact, preclinical studies show increased effectiveness due to the anti-viral immune response against oncolytic virotherapy for Newcastle disease virus, maraba, and reovirus, although the mechanisms are unknown [27,28,29]. Similarly, pre-existing antibodies did not affect the anti-tumor efficacy of an oncolytic Ad vector, INGN 007, after intra-tumoral administration in immunocompetent animals (though it did in immunosuppressed mice) [30,31]. Furthermore, in a Syrian hamster model, which is both immunocompetent and permissive for human Ad5 replication [30,31,32,33,34,35], it was observed that pre-existing immunity reduced off-target vector spread [30]. Potential explanations of why anti-viral immunity may be paradoxically beneficial include: (1) adjuvant-like properties of anti-viral innate responses prime for an anti-tumor immune response, or (2) immune responses to the virus within the tumor supports the recruitment of anti-tumor effector immune cells [36].

When reflecting on the clinical tumor regressions in select brain tumor patients, it is worth noting that these regressions occurred months after OV administration, when no active viral replication was detectable. Furthermore, the tumors initially increased in size, similar to the pseudo-progression observed after other immunotherapy treatments [37]. Lastly, regressions occurred just as frequently in the adenovirus trials as the others, even though ~70% of the adult population in the US is seropositive to Ad5 [38]. Pre-existing anti-Ad5 antibodies should have presented a serious hindrance to the clinical application of CRAds, but this was not the case, suggesting that pre-immunization did not preclude therapeutic efficacy after all. Notably, a recent landmark study showed that repetitive systemic OV dosing led to durable responses in several patients with cervical cancer [39,40].

#### 3.3.3. Immunogenicity of NSC.CRAd-S-pk7

Our study differs from others in that we are using a cell delivery vehicle that is not necessarily inert with respect to how the immune system recognizes and clears the virus, or how it interacts with the host immune system. Our NanoString transcriptome analysis suggests that, in general, the immune changes that occur in response to NSC.CRAd-S-pk7 treatment seem to be driven by the virus itself, as only minor fluctuation is seen when delivered via NSC. This finding is consistent with our previous findings that NSCs are generally immune-privileged, characterized by lack of major histocompatibility complex class 2 (MHC-II) expression and low MHC class 1 (MHC-I) expression [9], and thought to be poorly recognized within human leukocyte antigen (HLA) incompatible hosts. In a recent phase I trial of administering multiple intracerebral doses of HB1.F3.CD21 NSCs, only 3 of the 14 tested patients treated with intracranial NSCs developed anti-NSC antibodies after the third dose. No anti-NSC antibodies were detected with the other 11 tested patients, even after receiving as many as 10 doses of NSCs. Furthermore, no evidence of anti-NSC T-cell responses or immune-mediated toxicity was observed in study patients [41]. We acknowledge that CRAd infection may alter these favorable immunogenicity results by inducing the expression of stress antigens that serve as markers for the elimination of virally infected cells (by NK cells, NK T cells, gamma-delta T cells, and macrophages). Thus, the possibility an anti-NSC.CRAd-S-pk7 response developing will be monitored in our upcoming trial.

#### 3.3.4. Study Limitations

Our immediate translational objective restricted our study to a dose-equivalent to 150 million NSC.CRAd-S-pk7s (1.875 × 10^11^ Viral Particles). In a study of one vs. six cycles of measles virotherapy for ovarian cancer [42], multiple cycles were only advantageous at lower doses. While our study demonstrates improved treatment efficacy after three treatment cycles, it is possible that, at higher doses, no benefit would be seen. Furthermore, long-term repeat dosing regimens might eventually result in no additional therapeutic benefit. The present study also does not address the optimal frequency for repeated dosing. An interval of 1 week was selected in both preclinical and clinical settings to allow sufficient time for an adaptive immune response to occur in response to each successive treatment. Lastly, these IND-enabling studies assume that the basic features of the immune response to NSC.CRAd-S-pk7 treatments are similar in mice and humans [43]. While this assumption is generally valid for acute viral infections, there are some notable differences, particularly in FoxP3 and CD4 expression levels [44].

## 4. Conclusions

These IND-enabling studies set the foundation for a phase I study of intracerebral administration of multiple-round stem cell-based oncolytic therapy in high-grade glioma patients. This NSC-CRAd-S-pk7 treatment regimen may also enhance the therapeutic efficacy of other anti-glioma oncolytic virotherapies—each successive NSC.CRAd-S-pk7 administration serves to (1) attract further immune surveillance to the otherwise cold tumor microenvironment and (2) alert the immune system with updated information regarding tumor adaptations. Future work will focus on maximizing activation of anti-tumor immunity, perhaps through co-administration with checkpoint inhibitors or rapamycin, or in conjunction with anti-GBM CAR-T/NK cell treatments.

## Figures and Tables

**Table 1:** Explanation of Blood Chemistry Test Analytes.

Acronym	Analyte	Pathologic Association
ALT	Alanine Aminotransferase	Heart and Liver (viral hepatitis/cirrhosis) disease
ALB	Albumin	Liver and Kidney disease
ALP	Alkaline phosphatase	Liver, Bone, Parathyroid, and Intestinal disease
AMY	Amylase	Kidney and Pancreatic disease
CA	Calcium	Parathyroid, Bone, chronic Renal disease, Tetanus
CRE	Creatinine	Renal disease
GLOB	Globulin	Dehydration, Antigenic stimulation
GLU	Glucose	Diabetes, Hyperglycemia, Hypoglycemia, Liver disease
PHOS	Phosphorus	Kidney Disease, Hypoparathyroidism, Nutritional disorders
Na+	Sodium	Malnutrition, Renal disease, Vomiting, Diarrhea, Cardiac symptoms.
TBIL	Total Bilirubin	Hepatic disorders
TP	Total Protein	Dehydration, Kidney and Liver disease, Metabolic and Nutritional disorders
BUN	Blood Urea Nitrogen	Liver and Kidney disease

## Data Availability

The data presented in this study are available on request from the corresponding author.

## Supplementary Materials

The following are available online at https://www.mdpi.com/article/10.3390/cancers13246320/s1, Figure S1: Multiple NSC.CRAd-S-pk7 administrations in immunodeficient nu/nu mice.

## References

[1] Alemany R. (2012). Viruses in cancer treatment. Clin. Transl. Oncol..

[2] Ahmed A.U., Thaci B., Tobias A.L., Auffinger B., Zhang L., Cheng Y., Kim C.K., Yunis C., Han Y., Alexiades N.G. (2013). A Preclinical Evaluation of Neural Stem Cell–Based Cell Carrier for Targeted Antiglioma Oncolytic Virotherapy. J. Natl. Cancer Inst..

[3] Chiocca E.A., Abbed K.M., Tatter S., Louis D.N., Hochberg F.H., Barker F., Kracher J., Grossman S.A., Fisher J.D., Carson K. (2004). A Phase I Open-Label, Dose-Escalation, Multi-Institutional Trial of Injection with an E1B-Attenuated Adenovirus, ONYX-015, into the Peritumoral Region of Recurrent Malignant Gliomas, in the Adjuvant Setting. Mol. Ther..

[4] Kaufmann J.K., Nettelbeck D.M. (2012). Virus chimeras for gene therapy, vaccination, and oncolysis: Adenoviruses and beyond. Trends Mol. Med..

[5] Brunetti-Pierri N., Palmer D.J., Beaudet A.L., Carey K.D., Finegold M., Ng P. (2004). Acute Toxicity After High-Dose Systemic Injection of Helper-Dependent Adenoviral Vectors into Nonhuman Primates. Hum. Gene Ther..

[6] Kanojia D., Balyasnikova I.V., Morshed R.A., Frank R.T., Yu D., Zhang L., Spencer D.A., Kim J.W., Han Y., Yu D. (2015). Neural Stem Cells Secreting Anti-HER2 Antibody Improve Survival in a Preclinical Model of HER2 Overexpressing Breast Cancer Brain Metastases. Stem Cells.

[7] Mooney R., Majid A.A., Batalla J., Annala A., Aboody K.S. (2017). Cell-mediated enzyme prodrug cancer therapies. Adv. Drug Deliv. Rev..

[8] Mooney R., Hammad M., Batalla-Covello J., Majid A.A., Aboody K.S. (2018). Concise Review: Neural Stem Cell-Mediated Targeted Cancer Therapies. Stem Cells Transl. Med..

[9] Ahmed A.U., Thaci B., Alexiades N.G., Han Y., Qian S., Liu F., Balyasnikova I.V., Ulasov I., Aboody K.S., Lesniak M.S. (2011). Neural Stem Cell-based Cell Carriers Enhance Therapeutic Efficacy of an Oncolytic Adenovirus in an Orthotopic Mouse Model of Human Glioblastoma. Mol. Ther..

[10] Tobias A.L., Thaci B., Auffinger B., Rincón E., Balyasnikova I.V., Kim C.K., Han Y., Zhang L., Aboody K.S., Ahmed A.U. (2013). The Timing of Neural Stem Cell-Based Virotherapy Is Critical for Optimal Therapeutic Efficacy When Applied with Radiation and Chemotherapy for the Treatment of Glioblastoma. Stem Cells Transl. Med..

[11] Sonabend A.M., Ulasov I.V., Han Y., Rolle C.E., Nandi S., Cao D., Tyler M.A., Lesniak M.S. (2008). Biodistribution of an oncolytic adenovirus after intracranial injection in permissive animals: A comparative study of Syrian hamsters and cotton rats. Cancer Gene Ther..

[12] Lesniak M.S. Neural Stem Cell Based Virotherapy of Newly Diagnosed Malignant Glioma. ClinicalTrials.gov.

[13] Fares J., Ahmed A.U., Ulasov I.V., Sonabend A.M., Miska J., Lee-Chang C., Balyasnikova I.V., Chandler J.P., Portnow J., Tate M.C. (2021). Neural stem cell delivery of an oncolytic adenovirus in newly diagnosed malignant glioma: A first-in-human, phase 1, dose-escalation trial. Lancet Oncol..

[14] Priceman S.J., Tilakawardane D., Jeang B., Aguilar B., Murad J.P., Park A.K., Chang W.C., Ostberg J.R., Neman J., Jandial R. (2018). Regional Delivery of Chimeric Antigen Receptor–Engineered T Cells Effectively Targets HER2^+^ Breast Cancer Metastasis to the Brain. Clin. Cancer Res..

[15] Russell S.J. (2018). For the Success of Oncolytic Viruses: Single Cycle Cures or Repeat Treatments? (One Cycle Should Be Enough). Mol. Ther..

[16] Kim S.U. (2004). Human neural stem cells genetically modified for brain repair in neurological disorders. Neuropathology.

[17] Kim S.U., Nakagawa E., Hatori K., Nagai A., Lee M.A., Bang J.H., Zigova T., Sanberg P.R., Sanchez-Ramos J.R. (2002). Production of Immortalized Human Neural Crest Stem Cells. Neural Stem Cells.

[18] Kim S.U., de Vellis J. (2009). Stem cell-based cell therapy in neurological diseases: A review. J. Neurosci. Res..

[19] Kim S.U., Nagai A., Nakagawa E., Choi H.B., Bang J.H., Lee H.J., Lee M.A., Lee Y.B., Park I.H. (2008). Production and Characterization of Immortal Human Neural Stem Cell Line with Multipotent Differentiation Property. Neural Stem Cells.

[20] Tirughana R., Metz M.Z., Li Z., Hall C., Hsu D., Beltzer J., Annala A., Oganesyan D., Gutova M., Aboody K.S. (2018). GMP Production and Scale-Up of Adherent Neural Stem Cells with a Quantum Cell Expansion System. Mol. Ther.-Methods Clin. Dev..

[21] Danaher P., Warren S., Dennis L., D’Amico L., White A., Disis M.L., Geller M.A., Odunsi K., Beechem J., Fling S.P. (2017). Gene expression markers of Tumor Infiltrating Leukocytes. J. Immunother. Cancer.

[22] Zhang L., Hedjran F., Larson C., Perez G.L., Reid T. (2014). A novel immunocompetent murine model for replicating oncolytic adenoviral therapy. Cancer Gene Ther..

[23] Gutova M., Flores L., Adhikarla V., Tsaturyan L., Tirughana R., Aramburo S., Metz M., Gonzaga J., Annala A., Synold T.W. (2019). Quantitative Evaluation of Intraventricular Delivery of Therapeutic Neural Stem Cells to Orthotopic Glioma. Front. Oncol..

[24] Appledorn D.M., McBride A., Seregin S., Scott J.M., Schuldt N., Kiang A., Godbehere S., Amalfitano A. (2008). Complex interactions with several arms of the complement system dictate innate and humoral immunity to adenoviral vectors. Gene Ther..

[25] Miller A., Nace R., Steele M., Bailey K., Peng K.W., Russell S.J. (2016). Perfusion Pressure Is a Critical Determinant of the Intratumoral Extravasation of Oncolytic Viruses. Mol. Ther..

[26] Bailey K., Kirk A., Naik S., Nace R., Steele M.B., Suksanpaisan L., Li X., Federspiel M.J., Peng K.-W., Kirk D. (2013). Mathematical Model for Radial Expansion and Conflation of Intratumoral Infectious Centers Predicts Curative Oncolytic Virotherapy Parameters. PLoS ONE.

[27] Ricca J.M., Oseledchyk A., Walther T., Liu C., Mangarin L., Merghoub T., Wolchok J.D., Zamarin D. (2018). Pre-existing Immunity to Oncolytic Virus Potentiates Its Immunotherapeutic Efficacy. Mol. Ther..

[28] Niemann J., Woller N., Brooks J., Fleischmann-Mundt B., Martin N.T., Kloos A., Knocke S., Ernst A.M., Manns M.P., Kubicka S. (2019). Molecular retargeting of antibodies converts immune defense against oncolytic viruses into cancer immunotherapy. Nat. Commun..

[29] Conrad D.P., Tsang J., MacLean M., Diallo J.-S., Le Boeuf F., Lemay C.G., Falls T., Parato K.A., Bell J.C., Atkins H.L. (2013). Leukemia Cell-Rhabdovirus Vaccine: Personalized Immunotherapy for Acute Lymphoblastic Leukemia. Clin. Cancer Res..

[30] Dhar D., Spencer J.F., Toth K., Wold W.S.M. (2009). Effect of Preexisting Immunity on Oncolytic Adenovirus Vector INGN 007 Antitumor Efficacy in Immunocompetent and Immunosuppressed Syrian Hamsters. J. Virol..

[31] Thomas M.A., Spencer J.F., Toth K., Sagartz J.E., Phillips N.J., Wold W.S. (2008). Immunosuppression Enhances Oncolytic Adenovirus Replication and Antitumor Efficacy in the Syrian Hamster Model. Mol. Ther..

[32] Lichtenstein D.L., Spencer J.F., Doronin K., Patra D., Meyer J.M., Shashkova E.V., Kuppuswamy M., Dhar D., Thomas M.A., Tollefson A.E. (2009). An acute toxicology study with INGN 007, an oncolytic adenovirus vector, in mice and permissive Syrian hamsters; comparisons with wild-type Ad5 and a replication-defective adenovirus vector. Cancer Gene Ther..

[33] Thomas M.A., Spencer J.F., La Regina M.C., Dhar D., Tollefson A.E., Toth K., Wold W.S. (2006). Syrian Hamster as a Permissive Immunocompetent Animal Model for the Study of Oncolytic Adenovirus Vectors. Cancer Res..

[34] Ying B., Tóth K., Spencer J.F., Meyer J., Tollefson A.E., Patra D., Dhar D., Shashkova E.V., Kuppuswamy M., Doronin K. (2009). INGN 007, an oncolytic adenovirus vector, replicates in Syrian hamsters but not mice: Comparison of biodistribution studies. Cancer Gene Ther..

[35] Spencer J.F., Sagartz J.E., Wold W.S.M., Tóth K. (2009). New pancreatic carcinoma model for studying oncolytic adenoviruses in the permissive *Syrian hamster*. Cancer Gene Ther..

[36] Gujar S., Pol J.G., Kim Y., Lee P.W., Kroemer G. (2018). Antitumor Benefits of Antiviral Immunity: An Underappreciated Aspect of Oncolytic Virotherapies. Trends Immunol..

[37] Lang F.F., Conrad C., Gomez-Manzano C., Alfred Yung W.K., Sawaya R., Weinberg J.S., Prabhu S.S., Rao G., Fuller G.N., Aldape K.D. (2018). Phase I Study of DNX-2401 (Delta-24-RGD) Oncolytic Adenovirus: Replication and Immunotherapeutic Effects in Recurrent Malignant Glioma. J. Clin. Oncol..

[38] Stephenson K.E., Hural J., Buchbinder S.P., Sinangil F., Barouch D.H. (2012). Preexisting Adenovirus Seropositivity Is Not Associated With Increased HIV-1 Acquisition in Three HIV-1 Vaccine Efficacy Trials. J. Infect. Dis..

[39] Hotte S.J., Lorence R.M., Hirte H.W., Polawski S.R., Bamat M.K., O’Neil J.D., Roberts M.S., Groene W.S., Major P.P. (2007). An Optimized Clinical Regimen for the Oncolytic Virus PV701. Clin. Cancer Res..

[40] Zamarin D., Palese P. (2012). Oncolytic Newcastle disease virus for cancer therapy: Old challenges and new directions. Future Microbiol..

[41] Portnow J., Badie B., Blanchard M.S., Kilpatrick J., Tirughana R., Metz M., Mi S., Tran V., Ressler J., D’Apuzzo M. (2021). Feasibility of intracerebrally administering multiple doses of genetically modified neural stem cells to locally produce chemotherapy in glioma patients. Cancer Gene Ther..

[42] Peng K.-W., Hadac E.M., Anderson B.D., Myers R., Harvey M., Greiner S.M., Soeffker D., Federspiel M.J., Russell S.J. (2006). Pharmacokinetics of oncolytic measles virotherapy: Eventual equilibrium between virus and tumor in an ovarian cancer xenograft model. Cancer Gene Ther..

[43] Le D., Miller J.D., Ganusov V.V. (2015). Mathematical modeling provides kinetic details of the human immune response to vaccination. Front. Cell. Infect. Microbiol..

[44] Bjornson-Hooper Z., Fragiadakis G.K., Spitzer M.H., Madhireddy D., McIlwain D., Nolan G.P. (2019). A comprehensive atlas of immunological differences between humans, mice and non-human primates. bioRxiv.

