# Supplementary material for: Multiple Treatment Cycles of Neural Stem Cell Delivered Oncolytic Adenovirus for the Treatment of Glioblastoma
Source: Cancers (Basel). 2021 Dec 16;13(24):6320. doi: 10.3390/cancers13246320 (PMC8699772; doi:10.3390/cancers13246320)
Supplement: Supplementary file 1 [file cancers-13-06320-s001.zip › cancers-1471738-supplementary.pdf]

cancers

Multiple Treatment Cycles of Neural Stem Cell Delivered Oncolytic Adenovirus for the Treatment of Glioblastoma

Jennifer Batalla-Covello [1], Hoi Wa Ngai [1], Linda Flores [1], Marisa McDonald [1], Caitlyn Hyde [1], Joanna Gonzaga [1], Mohamed Hammad [1], Margarita Gutova, Jana Portnow [2], Tim Synold [3], David T. Curiel [4], Maciej S. Lesniak [5], Karen S. Aboody [1,*] and Rachael Mooney [1,*]

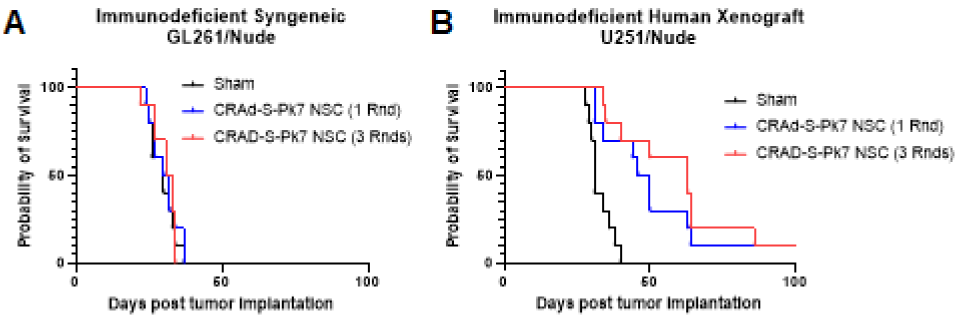

Figure S1. Multiple NSC.CRAd-S-pk7 administrations in immunodeficient nu/nu mice. (**A**) Immunodeficient nude mice implanted with GL261 tumors established using 2.0×10^3 cells/mouse, then treatment started Day 4 ($n = 10$ per group). Kaplan-Meier plot shows no survival benefit of either treatment regimen in this model. (**B**) Immunodeficient nude mice implanted with U251 tumors established using 5.0×10^4 cells/mouse, then treatment started on Day 3 ($n = 10$ per group). Kaplan-Meier plot shows no survival benefit, as Log-rank p-value is 0.2736 when comparing 1 vs. 3 treatments.
